# Mindfulness in Adolescent and Young Adult (AYA) Patients Undergoing Hematopoietic Stem Cell Transplantation (HSCT): A Qualitative Study

**DOI:** 10.3390/cancers14112760

**Published:** 2022-06-02

**Authors:** Sylvia L. Crowder, Rachel Sauls, Laura Redwine, Michael Nieder, Omar Albanyan, Hassaan Yasin, Farhad Khimani, Marilyn Stern

**Affiliations:** 1 Department of Health Outcomes and Behavior, Moffitt Cancer Center, Tampa, FL 33617, USA; sylvia.crowder@moffitt.org (S.L.C.); rsauls@usf.edu (R.S.); 2 Department of Psychology, College of Arts and Sciences, University of South Florida, Tampa, FL 33620, USA; 3 Department of Child and Family Studies, College of Behavioral and Community Sciences, University of South Florida, Tampa, FL 33612, USA; lredwine@usf.edu; 4 Department of Blood and Marrow Transplant and Cellular Immunotherapy, Moffitt Cancer Center, Tampa, FL 33612, USA; michael.nieder@moffitt.org (M.N.); omar.albanyan@moffitt.org (O.A.); hassaan.yasin@moffitt.org (H.Y.); farhad.khimani@moffitt.org (F.K.)

**Keywords:** mindfulness, quality of life, qualitative research, adolescent and young adult, cancer

## Abstract

**Simple Summary:**

Previous research in adolescent and young adult (AYA) patients suggests that patients undergoing hematopoietic stem cell transplantation (HSCT) experience severe physiological stress and social challenges. Therapeutic mindfulness interventions have been associated with improved well-being; however, we are unaware of any study that has examined AYA HSCT patients’ therapeutic interests in improving quality of life. The goal of this study was to identify unmet needs, interests, and preferences for mindfulness and quality of life prior to, immediately following, and three months post HSCT to inform the development of a mindfulness-based stress reduction intervention. Results suggest a mindfulness-based intervention may be beneficial in improving quality of life and enhancing social engagement, particularly immediately following HSCT. Future AYA research may benefit from employing mindfulness-based techniques to improve quality of life and psychosocial outcomes in HSCT patients.

**Abstract:**

Previous adolescent and young adult (AYA) research suggests patients undergoing hematopoietic stem cell transplantation (HSCT) experience severe physiological stress. The goal of this study was to identify unmet needs, interests, and preferences for mindfulness to inform the development of a mindfulness-based stress reduction intervention. Semi-structured interviews were conducted at three time points: prior to (*n* = 20), immediately after (*n* = 13), and three months post HSCT (*n* = 16) in the same AYA patients. Interviews assessed stress reduction strategies used, interest in mindfulness, and current quality of life. Three major thematic categories emerged from interview data across all time points: Concerns, Coping Strategies, and Mindfulness Activities. Prior to HSCT, two additional themes emerged including: Hope for the Future and Getting the Body Moving-Physical Activity. Most participants were not familiar with the term “mindfulness” prior to HSCT; but after being provided the definition of mindfulness, participants expressed interest in an online mindfulness-based intervention (e.g., ZOOM), stating: “I think it’s necessary” and “It would definitely be useful”. Participants suggested an intervention immediately following HSCT may decrease isolation concerns stating: “[in the hospital] You kind of feel like a hamster in a cage” and “you obviously have a lot of time to just be sitting by yourself in a hospital room”. The results suggest that a mindfulness-based online intervention is of interest to AYA HSCT patients and may be beneficial in decreasing physiological stress and improving quality of life.

## 1. Introduction

The National Cancer Institute defines adolescent and young adult (AYA) patients as 15–39 years of age [1]. AYA cancer patients have unique psychosocial needs [2] and historically experience a gap in healthcare services transitioning from pediatrics to adulthood that often affects these needs [3,4]. Hematopoietic stem cell transplantation (HSCT) is a potentially curative therapy for various aggressive and otherwise incurable malignancies such as relapse lymphomas, leukemias, and germ cell tumors [5]. Although AYA patients have noted improvements in outcomes recently, HSCT is an intensive process with many emotional, physical, and social challenges [4]. Prior to transplant, AYA recipients of HSCT often endure acute stressors related to the decision to undergo marrow transplantation, anticipated toxicities, financial burden, need for 24/7 caregivers, anticipated fertility loss, aggressive pre-transplant conditioning, and immunosuppression [6]. Immediately following transplant, loneliness, fear/anxiety, and perceived loss of control/depression often result from long isolation periods ranging from weeks to months post transplant [6,7,8,9,10]. After transplant, patients have a long period of adjustment before returning to their “new normal” life and are at increased risk of post-transplant complications, including infections, graft versus host disease, drug-related toxicities, organ toxicities, secondary malignancies, and other long-term effects up to two years and beyond [11]. To decrease these psychological stressors and improve emotional well-being, therapeutic interventions addressing psychosocial components of HSCT are warranted.

Over the past three decades, there has been a growing interest in using mindfulness-based interventions to improve quality of life [12]. Mindfulness interventions incorporate the state of awareness and consciousness while aiming to foster greater attention to the present moment and experience [12]. Methodologically rigorous studies and randomized clinical trials (RCT) have demonstrated that such interventions improve outcomes associated with pain, anxiety, and depression [12,13,14]. In a recent mindfulness-based intervention using the Headspace^®^ mobile app in *n* = 67 HSCT nurses, HSCT nurses reported significantly less burnout and improved well-being from baseline to every 30 days for 90 days with sleep hygiene meditations reported as the most widely used program [15]. In a pilot study of adult patients between 60 and 69 years with hematologic malignancies and their caregivers, participants rated baseline distress using the Rotterdam Symptom Checklist and were then taught how to complete a mind-body scan [16]. Two weeks later, participants reported increased relaxation, improved sleep, and showed interest in practicing mindfulness [16]. In non-HSCT populations, mindfulness-based interventions in AYA patients have been deemed feasible and acceptable [17], associated with improved quality of life and reduced emotional distress [18], and improved psychosocial well-being [19]. While these results are promising, we are unaware of any study that has examined AYA HSCT patients’ therapeutic preferences. Assessing preferences for this unique population is of particular importance as AYA patients are prone to quality-of-life issues during HSCT, resulting from dynamic life changes (e.g., moving out of their parents’ home for the first time, establishing career goals, forming social relationships) and symptomatology (e.g., fatigue, appearance concerns) [4]. Thus, the purpose of this qualitative study is to better understand the unique needs and preferences for mindfulness prior to, immediately after, and three months post HSCT in AYA patients. Specifically, quality of life, coping strategies, needs, and preferences were explored.

## 2. Materials and Methods

### 2.1. Ethical Approval

Protocol #Pro00037388 was approved by the Advarra Institutional Review Board of Moffitt Cancer Center and adhered to the principles of the Declaration of Helsinki. All participants were informed of the purpose and procedures of the study, and verbal informed consent was obtained from all prior to data collection.

### 2.2. Participants and Recruitment

This qualitative study consisted of English-speaking participants who were 18 to 39 years of age at study entry and eligible for HSCT as per the treating transplant physician. The treating physician and research coordinator arranged for the recruiting and consenting of participants using purposive sampling. A total of 24 AYAs were screened for the study and deemed eligible, 22 provided consent, and 20 completed the interviews (reasons for withdrawal included: (1) inability to conduct baseline interview prior to the treatment start date, and (2) personal health issues). Patients were required to have access to an iPad, iPhone, or computer with an Internet connection to participate in the ZOOM interviews. However, given the age of our population and increased use of remote delivery methods (e.g., ZOOM for work/school during the COVID-19 pandemic), Internet accessibility was not a limiting factor for the study. Interviews were scheduled based on participant convenience after discussion with the research coordinator. 

### 2.3. Data Collection

Participant demographics, disease, and transplant characteristics were extracted from electronic medical record reviews by the investigators (Table 1). A semi-structured interview guide was developed upon conduction of a literature review by psychologists and oncologists trained in mindfulness and HSCT (Supplementary Information). Interviews were conducted over three time points (prior to, immediately after, and three months post HSCT) to determine unmet needs, interests, preferences, and quality of life throughout the treatment trajectory. Semi-structured interviews were conducted over ZOOM by two researchers with training in qualitative interview methods between March 2020 and March 2022. Researchers were trained to provide only minimal verbal input and prompt only when appropriate during interviews [20]. Interviews were audio recorded, professionally transcribed verbatim, and checked for accuracy to ensure a complete account of participants’ responses. Probes allowed participants to voice concerns and unanticipated issues. 

### 2.4. Data Analysis

Data were analyzed using methods by Braun and Clark, a qualitative method for identifying, analyzing, and reporting themes, which allows for a rich, comprehensive description of events [21]. The first and second authors reviewed and read each transcript individually, then conducted line-by-line coding for statements related to mindfulness, preferences, coping, unmet needs, and quality of life independently. Codes were amended and refined through discussion until a single list of codes were agreed upon. The second author entered the list of codes in Dedoose^®^, a web application used for qualitative data analysis, and the first and second authors coded all transcripts, adding codes when necessary [22]. Discrepancies were resolved through discussion. Once coding was agreed upon and complete, the two authors reviewed the professionally transcribed coded transcripts to search for common themes to group codes together. These themes were reviewed, refined, and named until a finalized codebook was created (Table 2). Care was given to guard participant anonymity. 

## 3. Results

### 3.1. Descriptive Results

Table 1 displays the demographic and clinical characteristics of the study population. Participants, on average, were 29 years old, 75% of patients were non-Hispanic, and 45% were female. Indications for HSCT in included patients were Hodgkin lymphoma (30%), B-cell acute lymphoblastic leukemia (20%), and acute myeloid leukemia (20%). For 85% of participants, this was their first transplant. 

### 3.2. Qualitative Results—Thematic Findings

**Figure 1:**
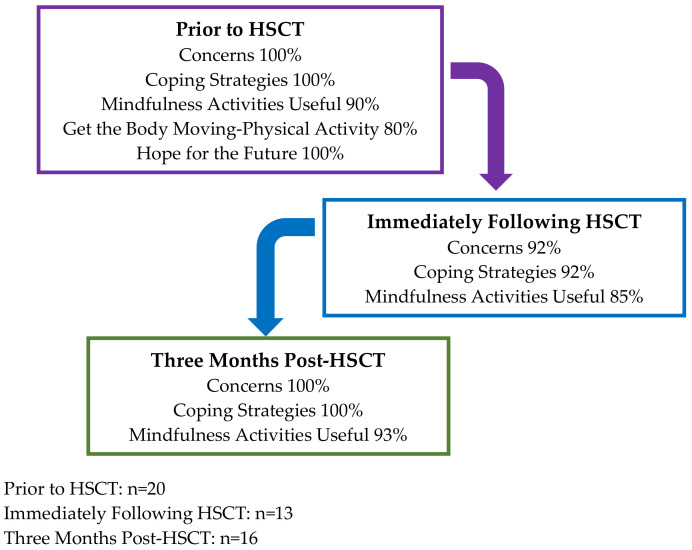
Percentage of AYA’s who stated common themes in qualitative interviews.

At each timepoint (prior to, immediately after, and three months post HSCT), three themes related to mindfulness, preferences, and unmet needs were derived from the semi-structured interview transcripts, including “Concerns”, “Coping Strategies” and “Mindfulness Activities”. In addition to these themes across each timepoint, timepoint 1 (prior to HSCT) had two additional themes occur, including “Hope for the Future” and “Getting the Body Moving-Physical Activity”. Headlines for the themes are presented in Figure 1. 

#### 3.2.1. Timepoint 1: Prior to HSCT

##### Concerns

Most participants expressed “anxiety”, “nervousness”, and “worry” about their upcoming treatment stating, “It was very nerve wrecking, going in blind, hearing all these horror stories about what could happen, and just everything. Trying to take in all the information… I’m still pretty nervous” (0012) and “I mean, I think I have what I think it’s gonna be like. And then I feel like I also have more of a worser-case scenario where I hope that’s not that way” (0022).

Several participants were parents of small children and expressed reservations about leaving their children for an extended period with family/friends stating, “But, like, I have to get better. I’ve got two kids. I don’t really have time for this, but we’re gonna figure out the time for it” (0013). Another participant stated, “I’m a mom, a young mom with two children so the past two weeks it’s really hit me emotionally, physically, and more now. I’ve been exhausted, I think, just mentally it’s draining me” (0006). While another said: “Yeah, now I’ve been on both ends. I’ve been the kid with the parent that’s sick, and now I’m the parent with the kid, and I’m sick” (0010).

Fear of isolation after treatment was a common concern, with participants stating: it would be “boring” (0015), “I’m assuming I’m probably gonna be bored out of my mind—17 days in the hospital” (0017), “the hospitalization, the only thing is it’s so hard to judge time” (0014), “I’d just say boring—it’s gonna be tough…” (0009), and “so that’s been the worst part, just thinking about sitting and being in the hospital for that long [30 days]” (0007). 

##### Coping Strategies

Participants reported developing a variety of coping skills, including FaceTiming friends and family and engaging in activities to keep them busy at home and in the hospital (e.g., coloring books, movies): “I try to see my friends and occupy my time. Just fill my life with my day-to-day stuff and not try to think too much about [the cancer]” (0022), “I talk to family… my dad is who I go to if I’m going to feel in the worst way. But really, just my family. But if I need somebody, I call my dad” (0018), “Sitting playing video games or playing guitar or doing something … I just try to stay productive throughout the day” (0019), “Netflix, Hula. I’ll color. I’ll read pretty much anything. So that kinda helps me not feel so anxious about things” (0016), “I’d say my social network’s pretty strong. I have a lot of friends that check up on me all the time. And family is really good as well” (0022). 

Participants also reported joining social networking groups on Facebook and Instagram to gain a sense of community and see the experiences of individuals their own age who had similar cancers: “[Social networking platforms] … these things that I’m in are thousands of people, and people of different ages, and I think if it was a smaller community where you could build a relationship with people… to some extent. The problem with stuff like that [social networking] is at the end of the day, it’s still a stranger, so—your friends know you, and you want to be able to talk to them. They don’t understand this no matter how hard they try, and I hope they never understand anything like this, but—I don’t know. I feel like it’s hard for me and probably other people—I’m not just gonna talk to a stranger and feel like ‘Yes, I’ve had a rough week, and I’m feeling emotional,’ and this and that. So, to some extent, yes, but also, it wouldn’t be anything like immediate gratification, like ‘Oh, I feel better, I vented, and now it’s off my chest’” (0011). 

##### Mindfulness Activities 

When our AYA participants were asked if they had heard of the term mindfulness, the majority of participants (60%) were not aware of the term responding “You know, I have not” (0022), “no, ma’am” (0021, 0017), “I’ve heard of the word, being mindful, but not the [definition]—yeah” (0010), “I guess, but not really” (0019), “no, you would have to educate me on it” (0018), “no” (0016, 005, 0012), “no idea” (0007), and “I don’t know” (0009, 0002). However, after the term was defined for them, all participants responded that they were familiar with mindfulness activities (ex. tapping, meditation) and interested in mindfulness (100%), suggesting it would be a beneficial tool to decrease stress. Participants stated that a mindfulness intervention would be “pretty dope” (0010) and “it sounds cool” (0019), stating, “I think it would be a pretty good helping tool. I do have anxiety myself… it would be very helpful to know other coping mechanisms and things like that” (0020), and “I would say it’s something I’m very interested in and definitely want to work on my skills building that” (0011) with 90% reporting that mindfulness-based interventions would be a useful strategy to reduce stress before HSCT. 

When participants were asked what type of mindfulness-based activities they currently do, if any, the majority responded that they weren’t currently doing activities they would consider mindful, but for the few who responded, they stated: “I used to do yoga” (0014), “I really enjoy yoga. When I do yoga, and I’m trying to do deep breathing, and meditate, and channel thoughts to one central thing, that’s what I think of” (0013), “Journaling… I do the Calm app and sometimes I’ll do little meditations on YouTube or Calm” (0011).

##### Hope for the Future

Despite concerns AYAs had regarding treatment, they often used positive thinking to improve their mindset and tried to avoid thinking about their cancer, diagnosis, and treatment, stating, “I look at the Brightside” (0018), “I’m optimistic, trying to look on the positive side of things” (0016), “… not think of anything bad… I just try to look at everything positive” (0003), “just gotta think of the future” (0004), “I feel really, really good. Physically and mentally and emotionally, which is the main thing. I’m feeling very good about this, about the transplant and the whole process of it” (0020), and “I’ve never been a Debbie downer kinda person. So, I always try to look at the positive side of things. My whole life, I was one of those types of people who didn’t let it affect me in my way of living. ‘Cause some people use that as an excuse. And I just think it was all really mind over matter” (0019). 

A handful of participants avoided thinking about their cancer diagnosis and treatment and used humor as a coping mechanism: “I don’t like to think about having cancer… I crack a lot of jokes. That’s how me and my mother cope with the cancer thing. When I got here, I did my edges to stunt on these bald bitches, but that’s just my humor” (0010). 

Several participants discussed the future in terms of “getting back to normal” and returning to a pre-diagnosis life, stating, “I mean ideally, life goes back to normal… I’d like to say that after this, you know, a couple months from now I’ll be getting back to my normal life” (0005), “I’m seeing a future wherein I can be back in my normal life working, taking care of my husband, supporting my family…” (0021), “I just want to put it all behind me, look forward to good health” (0017), “It’s gonna be a lot better. I can be more independent than now” (0015) and “I’m really hoping that I can be back to normal” (0013). One participant stated: “My hope is that I am back to normal… My hope is that life is the way it used to be… I think that as long as I do my part and I stay optimistic about it all, that it will hopefully be as close to what I think it should be/want it to be. That’s it. So, my goal is after the transplant I will be back to normal” (0022).

##### Getting the Body Moving-Physical Activity 

Before transplant, there was no lack of motivation for physical activity, with 80% reporting engagement in some physical activity; however, participants noted several perceived barriers to continuing with physical activity after transplant, including lack of energy/fatigue, nausea, tubes/ports, pain, worry about “overdoing it”, and lack of variety while in the hospital with participants stating “it’s just one hallway in the hospital… I need some variety” (0011). Participants expressed, “I was definitely a lot more physical before than I am [now]” (0007), “[no barriers] nothing, ma’am. Just this condition that I have because of the port so I have to minimize my movements” (0021), “So, I have a nephrostomy tube… And that’s kind of a barrier to do too much physical activity. Yeah. So, without that—I’m sure you would get fatigued a little faster. But nothing that would stop you at least from doing more physical activity” (0014), and “The only normal I feel like I’m not doing right now is the exercise part. Otherwise, I feel like my life hasn’t been—I mean, I’m still working. I’m still doing stuff. I’d like to get back to the normal of exercise” (0013). 

Some participants expressed concerns regarding physical activity and how it has impacted their family/home life: “Honestly, I would love to be able to walk again, longer than five minutes, without huffing and puffing.…I’m excited to actually be able to get up and move around again, especially as a mechanic. I don’t have time to kinda—I will literally work on something, and 10 min later, I gotta sit down and sit in front of a fan and then recoup, and then get up. I am so ready to actually be able to be physical again. I have two little girls. Do you know how hard it is to chase two little girls when you’re huffing and puffing? It’s not fun” (0009). While other participants discussed general concerns: “My problem has been the achiness, the nausea. Those have been things that limit me to walking around and stuff. But really, once I can get out and I’m alright like once I get rid of the nauseousness and just being out the hospital… but I’m still able to do stuff around my yard” (0007). 

#### 3.2.2. Timepoint 2: Immediately Following HSCT

##### Concerns

Immediately following transplant, participants reported feeling “nervous”, “anxious”, and “worried”. One participant shared … “the new cells not engrafting mostly. I think that’s the only thing I was really worried about” (0016) while another stated “It was a little nerve wracking, not knowing exactly what I’m… getting myself into… so it was a little nerve wracking” (0012). One participant reported hesitation around the treatment stating, “I’m a little skeptical until we do these scans and see that it worked” (0005). Concerns regarding fear of the future/unknown were commonly discussed immediately after treatment. Participants also expressed concerns regarding the cost of treatment “I thought that everything will be covered by the insurance and the stem cell. And then, now, I am just surprised that, because I became inpatient, I was just surprised that some of the fee is not covered by the insurance because it’s inpatient” (0021). 

Appearance was another common theme that arose immediately following treatment, with participants stating, “Having some skin rash issues everywhere right now…it seems to have spread more than yesterday” (0013), “different [pigment] colors and changes, and it’s very strange what’s happening with my body. And I’m definitely not happy about it” (0012), and “I don’t know if you guys noticed, but I don’t have hair anymore. So, as a female, not having hair is a pretty big deal, it gets to me sometimes” (0010). Isolation concerns were highest immediately following transplant, with participants stating, “I was in isolation so I couldn’t leave my room for three weeks” (0010), while another participant said, “You kind of feel like a hamster in a cage after a while” (0004). 

##### Coping Strategies

Similar to what was found prior to treatment, immediately after treatment, participants used positive thinking to avoid thinking about the negative consequences of transplant. One participant stated, “to me it’s really positive thinking… I think the biggest thing is just being very optimistic of what the transplant will bring” (0022), while others felt well informed regarding treatment decisions that boosted understanding and confidence “I was still pretty confident going in, just because I knew what was to be expected. And I mean emotionally, I really—I wasn’t too bad. I was pretty confident” (0012). Participants expressed using a variety of different strategies to stay busy and “distract myself” (0005) in the hospital, including “talking to friends, praying, having devotion” (0021), “sleeping” (0016), “movies and books, videos games, Netflix” (0015), and “coloring” (0013). Participants stated, “we knew that we were gonna get bored and we knew that there’s going to be a lot of downtime, so, we definitely prepared for that… crafts… a lot of movies… it did work as a coping mechanism” (0012). Several participants were able to work remotely and stated, “I definitely noticed the days went by fast during the week when I worked, then the weekend when you’re just kinda in the room for long periods of time. So, having meetings with people at work and things like that. It just kinda passed the time a lot quicker” (0014). Other participants wanted to use the hospitalization period for self-improvement after feeling like they were given a new chance at life and stated, “I’ve been more focused on broadening my horizons, like making myself a better person. I’ve been reading more, the books I’ve been reading are about empowerment. I just learned that insurance isn’t for old people. Like, if I get my daughter insurance now, she’ll be a millionaire by the time she’s my age. So, just books, learning new trades and skills and stuff like that. Because, you know, I’ve got a new chance at life. I wanna be better than I was before” (0010).

##### Mindfulness Activities 

Participants (85%) stated a mindfulness intervention immediately following HSCT would be a useful strategy for reducing stress, stating, “I think this is where I need it the most… I think it’s necessary” (0006) and “You obviously have a lot of time to just be sitting by yourself in a hospital room” (0013), “I would participate” (0003), “I would definitely try it… It would definitely be useful for me now” (0004), “I think meditation can be helpful. I think it’s good to be reflective” (0022), and “I think it would’ve been really nice to have that experience and to have somebody there helping you understand how to do some of that guided mediation and mindfulness thinking” (0013). A few participants (*n* = 2) felt that they would not benefit from mindfulness as they either didn’t feel a lot of stress or used other coping mechanisms (e.g., faith/prayer).

When participants were asked what type of mindfulness-based activities they currently do, they stated “the deep breathing and the imagery. They go through that as well in the Tapping Solution. So, I do those, too” (0010), and “Deep breathing strategies… mentally taking myself out of the hospital room and just thinking this is gonna pass” (0004). Participants felt a mindfulness-based intervention would be ideal as “I’m not very good at meditating. That’s why I think having somebody there to help with a guided mediation would be helpful” (0013). Specifically, participants were interested in deep breathing exercises, tapping, mediation exercises, and slow-moving/relaxing forms of yoga. 

#### 3.2.3. Timepoint 3: Three Months Post HSCT

##### Concerns

Three months post HSCT, participants discussed concerns regarding isolation; however, this feeling of isolation was different from the isolation experienced immediately following treatment. One participant shared, “So, it’ll take some time, but they said about two years… I have to get all my baby shots again, and my immune system is equivalent to a baby’s right now… I got a little virus that for most people, you won’t know you have it because you think it’s just allergies or something. But for me, it put me on my ass for like a week” (0019). Another participant stated they are “staying home 24/7” (0015), and another shared, “I’m still in an isolation… and after a while painting gets boring, video games get boring, watching TV… seeing social media and all my friends out having fun celebrating the holidays, like St. Patrick’s day… they were all going out and having a good time. And I’m sitting here recovering” (0012). 

##### Coping Strategies

Participants reflected on their time in the hospital immediately following treatment, stating, “I mean, when I was in the hospital it was basically just trying to sleep and recover and just get through it. That was the biggest thing it’s just—you know, focus on getting out and getting better” (0022). Others reflected on their cancer survivorship trajectory wondering what life would have been like had they never been diagnosed with cancer, “Yeah. I mean, it’s like those three years are just gone. You’re like all the time that I could have been in school or I could have been—I could have moved to Colorado for all we know. Those three years I can’t get back…” (0012). Participants shared the importance of trying to keep a positive mindset when you feel down because “your mind just plays a lot of tricks. And there was a moment where I was like, ‘Oh my god, am I going to make it? What happens if I don’t? My son will get cheated out of having his mom.’” (0020). Another participant shared, “you got to keep your mind busy while you’re in there… just keep optimistic and keep focused” (0004). 

##### Mindfulness Activities 

When discussing mindfulness, participants three months post HSCT stated, “it kind of helps you give you control” (0004). Another reflected immediately following treatment when isolated in the hospital, saying, “I wish I brought my yoga mat with me and done some yoga” (0013). Another stated, “anything physical, like, any, kind of, like, exercise. I think exercise would probably be the biggest” (0002). When participants were asked to reflect on their mindfulness preferences prior to, immediately after, and three months post HSCT, participants stated that a telehealth delivery of a mindfulness intervention would “be kind of cool” (0015) and a “useful” strategy to have in the hospitalization period (0014). One participant elaborated, “oh yeah, for sure. I feel like it would definitely be good in the hospital… I feel like if someone was encouraging you to do it, or maybe you schedule a time on ZOOM” (0011). Specifically, during the hospitalization period, when participants are away from family and friends and under a lot of stress would be ideal for implementing a mindfulness-based intervention “now that I’m with my daughter, I really don’t have any stress. In the hospital, I did” (0003). 

While 93% of participants felt mindfulness would be a useful strategy to reduce stress, one participant did not think a mindfulness intervention would benefit them and stated, “I don’t know, I guess I just lost interest in it, because again I didn’t do anything because I was out of it most of the time” (0016). There were also some contradictions in what mindfulness meant to participants. For instance, one participant expressed daily affirmations and words of gratitude as part of their everyday routine but did not think mindfulness would be helpful, stating, “Mindfulness? I just—mindfulness and meditation aren’t something I practice. Like I said, I always tell myself, ‘You’re doing good. You got this. Don’t beat yourself up over small things.’” (0006). 

## 4. Discussion

This qualitative study provided insight into the experience of AYA patients prior to, immediately following, and three months post HSCT. Thematic analysis suggests AYA HSCT patients have unmet needs regarding coping and anxiety/stress-related concerns throughout the treatment trajectory. Despite these concerns, many patients reported using positive thinking, humor, and mindfulness (e.g., tapping and meditation) to cope with uncertainty, and participants were particularly interested in deep breathing exercises, tapping, mediation exercises, and slow-moving/relaxing forms of yoga incorporated into a mindfulness intervention. Prior to HSCT, findings suggest a large proportion of AYA patients were not familiar with the term mindfulness; however, patients reported that a mindfulness-based intervention would be helpful in decreasing stress and improving quality of life. Participants reported a mindfulness-based intervention would be useful prior to HSCT (90%; *n* = 18), immediately following HSCT (85%, *n* = 11), and three months post HSCT (93%; *n* = 15). Of the participants who favored an intervention immediately following HSCT, most acknowledged isolation being a main factor for their decision, while the two participants who did not favor an intervention immediately following HSCT reported faith/prayer being their own forms of mindfulness. When comparing prior to HSCT to just immediately following, the common theme of “Hope for the Future” transitioned to survivors stating concerns with the unknown regarding survival and transplant success. Physical activity was also a common theme prior to HSCT; however, comments regarding physical activity and exercise were not commonly discussed by patients immediately following or three months post HSCT. This is likely a result of the interview protocol, as questions directly related to physical activity were only asked prior to HSCT; however, for the few participants who stated they remained physical active (15% immediately following treatment and 13% three months post treatment), they stated activity decreased resulting from increased fatigue and negative side-effects post transplant (e.g., decreased cardio capacity). 

To our knowledge, this is the first qualitative study conducted in AYA HSCT to examine unmet needs and preferences for a mindfulness-based intervention. Previous research has suggested that mindfulness-based interventions, when designed for caregivers of HSCT, improve post-traumatic growth and general mental health [23,24]. Furthermore, research has indicated that older HSCT patients (mean age 51–52 years) who participated in a mindfulness-based intervention reported improvements in quality of life, well-being [25], respiratory rates, and symptoms [26]. HSCT caregivers reported a preference for a mindfulness-based intervention during both the inpatient and outpatient phases with a combination of in-person and mobile-based delivery options [24]. In our AYA HSCT patients, concerns regarding isolation were common, specifically immediately following HSCT. Thus, participants expressed interest in a mindfulness intervention to improve social interaction, mental health, and well-being while reducing feelings of loneliness. Given concerns regarding participants’ physical safety and compromised immunity, AYA patients indicated a preference for an online (e.g., ZOOM) intervention and believed it would likely help improve quality of life and mental outlook.

In our study, participants reported using positive thinking, distracting themselves/staying busy, social networking, and mindfulness techniques to cope with treatment. In contrast to other passive therapeutic interventions (e.g., acupuncture, massage therapy), mindfulness requires active participation and, once learned, can be used without assistance. Thus, by taking a more active role in symptom management, participants may be increasing their perceived sense of self-efficacy and confidence in their ability to influence behavior change [27]. In this preliminary study, there were no differences in common themes when accounting for sex, age, or type of transplant received. However, most participants in our study (85%) received their first transplant and the lived experience of first transplant patients differed in comparison to participants who were receiving their second transplant. While the sample size was small (*n* = 3), participants receiving their second transplant felt better prepared for the isolation period reporting they “knew what was to be expected”. Additionally, two of the second transplant patients were already using meditation as a mindfulness activity prior to transplant. While the isolation period was not as draining for participants receiving their second transplant, these individuals still expressed interest in a mindfulness-based intervention and felt it would be a useful tool to pass the time and improve well-being. 

A strength of the present study is the use of repeated thematic interviews to explore changes in the HSCT trajectory. The same participants were interviewed prior to, immediately after, and three months post HSCT to gauge interest and preferences for mindfulness while identifying unmet needs and quality of life concerns. Repeated measures allowed the investigators to establish rapport with participants, encouraging a deeper exploration of their lived experiences and HSCT preferences. The interviews took place via ZOOM, providing participants with increased flexibility for scheduling as they did not need to make special arrangements to meet at the NCI-designated comprehensive cancer center. Finally, these findings are based on AYA HSCT patients’ own voices as they articulate their preferences and unmet needs. While the study has several strengths, limitations should be noted. The study was conducted during the COVID-19 pandemic; thus, isolation concerns could have been heightened as these participants were immunosuppressed. This study reflects a small sample of AYA HSCT patients treated in an urban setting; thus, results may not be generalizable to all AYA HSCT patients. However, HSCT is typically only available in large specialized centers, and our referral is from urban and rural communities. We were unable to blind the research question to participants; thus, it is possible that individuals who participated in the study were also most interested in learning about mindfulness-based interventions. While the goal of the current study was to assess interest in mindfulness and provides preliminary support for interest in mindfulness interventions in AYA HSCT patients, further feasibility research is warranted (e.g., pilot clinical trials) to best tailor the unique needs of AYA HSCT patients. Additionally, only a description of mindfulness was provided, and AYA HSCT patients did not get the opportunity to actually practice these exercises and provide feedback. Furthermore, it is possible that as a result of study participation, participants partook in their own mindfulness activities more so than they would have without the study. Unfortunately, we saw a drop in participant interviews from before to immediately following HSCT, primarily resulting from fatigue. This is important as AYA HSCT patients who completed interviews immediately following HSCT stated isolation concerns were heightened, and a mindfulness-based intervention to improve fatigue, coping, and reduce stress would be favorable (85%). Future studies should employ safeguards to promote participant engagement to improve quality of life immediately following the transplant process when interventions are likely needed resulting from isolation concerns. Another limitation of the study is that it only included English-speaking AYA HSCT patients; thus, future studies should incorporate additional languages such as Spanish-speaking AYA HSCT to better design culturally relevant and tailored interventions. These findings suggest that a mindfulness-based intervention is of interest for AYA HSCT patients, and such studies may improve quality of life and well-being by increasing social engagement during long isolation periods.

## 5. Conclusions

This study presents the first data on perceptions of a mindfulness-based intervention for AYA HSCT patients. Information on preferences, unmet needs, quality of life, and coping were collected. Participants reported that a mindfulness-based intervention would be favorable in improving quality of life. The results of the study suggest a mindfulness-based intervention conducted over an online platform, such as ZOOM, would be beneficial in decreasing physiological stress while creating a sense of community and engagement. These findings can guide the development of future mindfulness-based interventions in this population. 

## Figures and Tables

**Table 1:** AYA HSCT participant characteristics.

Participant Characteristics	*n* = 20 (%)
Age: mean ± SD [range], years	29.8 ± 5.6 [23.0–39.0]
BMI: mean ± SD [range]	28.4 ± 6.08 [17.5–45.0]
Sex: *n* (%) female	9 (45.0)
Ethnicity: *n* (%) non-Hispanic	15 (75.0)
Race: *n* (%)	
White	15 (75.0)
Black	2 (10.0)
Filipino	1 (5.0)
Other	2 (10.0)
Diagnosis: *n* (%)	
^a^ T-NHL	3 (15.0)
^b^ AML	4 (20.0)
Germ cell tumor	2 (10.0)
Hodgkin lymphoma	6 (30.0)
^c^ B-NHL	1 (5.0)
^d^ B-ALL	4 (20.0)
Conditioning chemo: *n* (%)	
Myeloablative	17 (85.0)
Reduced intensity	3 (15.0)
Conditioning: *n* (%)	
BEAM	7 (35.0)
FluBu5300	4 (20.0)
FluBu3500	2 (10.0)
FluMel	1 (5.0)
Carbo-VP16	2 (10.0)
CyTBI	2 (10.0)
FluTBI	2 (10.0)
Transplant number: *n* (%)	
First transplant	17 (85.0)
Second transplant	3 (15.0)
Transplant type: *n* (%)	
Autologous	9 (45.0)
Allogenic	11 (55.0)

^a^ T-cell non-Hodgkin lymphoma; ^b^ acute myeloid leukemia; ^c^ diffuse large B-cell non-Hodgkin lymphoma; ^d^ B-cell acute lymphoblastic leukemia.

**Table 2:** Themes and codes from semi-structured interviews of AYA HSCT patients.

Timepoint #1 (Prior to HSCT)		
Code	Description	Study ID, Patient’s Sex: Example Quote
**Theme 1: Concerns**		
Treatment anxiety	Nervousness, anxiety, worry, or stress	0003, Female: But. I’m nervous about the chemo, because they said it was 10 times stronger than my last one.
		0002, Male: I’m feeling nervous and scared. But I just want to get it over with.
Home responsibilities	Worried about home responsibilities including children	0004, Male: More scared for my son because he’s five and he doesn’t really grasp—that I’m gonna be away from a while… he gets home and he doesn’t see me, and then you know the day keeps on going, he’s not really, he’s not seeing me, he’s just seeing me through FaceTime.
		0010, Female: The most I’m worried about is my daughter. I have a one-year-old. So, being away from her for any amount of time is too much for me. But I’ve got to do what I’ve got to do.
Isolation	Any report of being alone, feeling lonely, or the need to connect with individuals of similar age	0017, Male: The only thing I’m worried about, like I said, is being bored and away from family and friends that long.
**Theme 2: Coping Strategies**		
Friends/family	Seek social support from friends or family members	0011, Female: My closest friend I would say just listens to me and I can just go on a tangent and it’s like nothing I say is too scary and she doesn’t get uncomfortable.
Activities to stay busy at hospital	Hospital activities	0003, Female: I got a bunch of stuff to take with me, like movies and coloring books.
		0011, Female: In the hospital… we watch a lot of Netflix and just stuff like that. Or I brought my switch and played Animal Crossing.
Activities to stay busy at home	Home activities	0011, Female: Yeah, I bake a lot at home. It’s like my core routine hobby. So, I feel like that’s been very therapeutic for me.
Social Networking	Interest or disinterest in social networking platforms	0004, Male: With other people that have been going through the same thing… yeah, I would be interested in it [social networks].
Extra activities	Medications, seeking professional help, etc.	0002, Male: Just cry it out.
		0011, Female: Well, I have Zoloft prescription and that helps a lot I feel like. And I just do things that I know help me. I will read or I write. I journal. I do a lot of that. And maybe like mindfulness stuff. Or if I can’t sleep, I use the calm app.
		0010, Female: I smoke a lot of weed.
**Theme 3: Hope for the Future**		
Positive mindset	Using positive thinking	0004, Male: And just gotta think of the future… it’s on my heart… My way of positive thinking is just thinking of the goals I want to do… because you know, be able to give other feedback on what worked, what didn’t work.
Avoidance of cancer thoughts	Avoid thinking of the treatment, cancer, or diagnosis	0003: Male: Not sitting at home is the best thing. Because then you think about everything [cancer].
Getting back to “normal”	Discussion of life returning to pre-diagnosis life	0004, Male: When I get back and just, this is what was placed in my hands to defeat… hopefully I’ll be able to do the things that I’ve been doing.
**Theme 4: Mindfulness Activities**		
Awareness of mindfulness	Discussion of being aware or unaware of the term mindfulness	0011, Female: I associate it, I guess, mainly with taking time to yourself to reflect and relax or ease your mind.
		0018, Male: No, you would have to educate me on it.
		0014, Male: Kinda perhaps meditation, or just being able to get into your own thoughts… something mindful, you know?
Tried mindfulness	Discussion of trying or not trying mindfulness activities	0011, Female: Yeah… I do the Calm app and sometimes I’ll do a little meditation on YouTube or Calm. It’s not every day, but I try to do it when I feel I need it.
Interest in mindfulness	Discussion of interest in mindfulness activities	0004, Male: Yoga. More chemo patients who are, maybe it’s a little slower, it’s a little—trying to do the stance, the praying mantis stance. You know? Stuff like that.
Barriers to mindfulness	Potential barriers to mindfulness activities	0002, Male: Yeah. It’s also the hardest here [hospital], I think. When I’m at home, or somewhere else, I can put this [cancer] away. But when I’m here [hospital], it’s in your face.
**Theme 5: Getting the Body Moving—Physical Activity**		
Motivation for physical activity pre-treatment	Motivation or lack of for physical activity	0022, Male: I really enjoy walking a lot. And that’s something that I found I like doing. I think it’s a good thing for your body. And I think it just gets you out there moving and everything.
		0013, Female: I want to be active… I have a two-year-old. I mean, there’s no getting around being active when there’s a two-year-old running around your house.
Motivation for physical activity pre-cancer	Activity level prior to cancer diagnosis	0002, Male: I usually do a lot of physical activities which I’ve had to cut back on.
Physical activity barriers	Barriers/challenges for physical activity	0004, Male: Like just walking to the restroom from the bed, is really challenging.
**Timepoint #2 (Immediately after HSCT)**		
**Code**	**Description**	**Study ID, Patient’s Sex: Example Quote**
**Theme 1: Concerns**		
Procedure anxiety	Description of feeling nervous or anxious before or during procedure	0016, Female: I was kind of nervous about the new cells not engrafting mostly. I think that’s the only thing I was really worried about
		0004, Female: … You never know what’s gonna happen when you have like tubes plugged into you
		0010, Female: I was a little anxious because I didn’t know how it was going to go.
Appearance	Discussion of appearance changes from cancer or procedure	0010, Female: Like today, I was a little upset because, I don’t know if you guys noticed, but I don’t have hair anymore. So, as a female, not having hair is a pretty big deal, it gets to me sometimes
		0012, Female: I’ve definitely noticed changes in my physical being, with my skin. On my face, I have got this pigment issue going on. It has become a little more prominent
Isolation	Feeling or not feeling isolated or bored in the hospital	0010, Female: Well, I was in isolation so I couldn’t leave my room for three weeks.
		0013, Female: I didn’t feel isolated at all. There were cards and things coming to the hospital every day and, again, people reaching out every day
Unknown future	Fear of the future—recurrence, comorbidities, negative health outcomes, etc.	0004, Female: you don’t really know how you’re gonna turn out because they talk about graft versus host disease, where can’t walk afterwards
		0005, Female: I don’t know what could happen, and if anything happens, I have to get rushed to a hospital
**Theme 2: Coping Strategies**		
Positive thinking	Using positive mindset during and after procedure	0003, Male: I just went in there as positive as I could, hoping for the best
Getting back to normal	Discussion of getting through the procedure to get back to normalcy, away from cancer	0004, Female: I just want to get it done and get back to whatever a normal life is these days
		0010, Female: there are a ton of rules, so I need to stay focused and heal to get better and back to normal
Staying busy	Discussion of staying busy and distracted while in hospital	0011, Female: I would say just trying to keep as busy as I could in the hospital. My mom and I would section off our time
		0015, Male: Watched Netflix, called family members, walked and talked to people, video games, movies, and books
Family/friends	Any mention of friends and family being a coping/relief during and after transplant	0005, Female: I had my husband by my side, it was very nice
		0010, Female: [Family/friends say] You’re beautiful, don’t worry about it. It’s just hair, it’s gonna grow back, girl. We’re here for you, God got you. They’ve been very supportive, very loving people. And I appreciate it.
**Theme 3: Mindfulness Activities**		
Mindfulness in hospital	Discussion of using mindfulness in hospital	0003, Male: [I] meditated in hospital with my step-mom, first time trying it. Meditated for about 15 min.
		0005, Female: I used the calm app to sleep some in the hospital because you’re constantly being woken up.
		0011, Female: My mom and I did a little mindfulness meditation on the TV screen when we were in the hospital, and I just had a scan the other day, and I listened to a meditation when I was waiting before because I get stressed.
Useful strategy	Timing of mindfulness to be most useful	0012, Female: Probably [immediately] after, just because during the transplant, there’s a lot that goes on. And there’s a lot—I mean, when you’re there—I mean, you get—I mean, it’s daily, you get people coming in.
		0010, Female: [I] would say it’s very useful because the—well, not me, because you know I’m awesome—but the anxiety that you get going into something that you really don’t know much about, like bone marrow transfusion
Interest in mindfulness	Interest or disinterest in mindfulness	0013, Female: I wish you guys were already doing the program because I do think it would’ve been helpful during to do some kind of guided program where somebody was actually kind of talking you through how to stay in the moment, especially leading up to the transplant.
		0014, Male: I’d say it’s useful, yeah, just being aware of staying focused on what you can handle at the given time and not, like you said, if it’s good or bad.
Improve engagement	Discussion of ways to improve engagement and attendance in a mindfulness program	0012, Female: I guess just stats to see how—just to knowing how it goes better, other people and if there were doctors who had said, “Yeah, this percentage of people are more successful in their healing, than these people who didn’t.”
**Timepoint #3 (Three Months Post HSCT)**		
**Code**	**Description**	**Study ID, Patient’s Sex: Example Quote**
**Theme 1: Concerns**		
Post-hospitalization anxiety	Description of feeling nervous or anxious post procedure	0005, Male: Honestly, yes. A little uncertainty. I mean, driving on the way here, my neck started hurting a little bit and I started feeling around and I felt like a small lymph node.
		0013, Male: Good. I mean, it was getting rough the last couple of weeks before I went home. I think that was the hardest. The two hard periods, I think were days three to 11 when I had mucositis really bad because I couldn’t really talk much, and it was painful and you’re by yourself at the hospital and that was rough.
Isolation after discharge	Feeling or not feeling isolated or bored after discharge	0011, Male: It was suffocating almost just because it’s like you can’t go anywhere or do anything. I mean, the pandemic is partially because of that. But yeah, and I just felt uncomfortable walking around, outside the hotel just because I was like people aren’t wearing their masks outside and which, I guess it’s okay. But I didn’t feel safe, just because I’m a newborn basically. So, it was just weird. Weird is the best way to put it. Yeah.
		0002, Male: Yeah. So, like, most—cause I stayed at a hotel, and I, kind of, got, not lazy, but, like, I didn’t do much at all. So, I just, like, felt miserable, so I was just, like, stayed miserable. So, I wish I would have, like, kind of, tried to do—be a little more active, I guess.
Unknown future after discharge	Fear of the future— recurrence, comorbidities, COVID-19 fears, and negative health outcomes after hospitalization	0019, Male: It was a painful three months of being in the hospital, and then having to stay local for a while. I was always worried about it, but it did its job.
		0020, Female: Oh my god, am I going to make it? What happens if I don’t? My son will get cheated out of having his mom.
**Theme 2: Coping Strategies**		
Positive thinking after discharge	Using a positive mindset after hospitalization	0006, Female: Like I said, I always tell myself, “You’re doing good. You got this. Don’t beat yourself up over small things.”
		0007, Male: Just trying to stay positive and just trying to look at the end of the tunnel or the end of the road.
Getting back to normal after discharge	Discussion of getting through the treatment to get back to normalcy, away from cancer post hospitalization	0005, Male: Pretty good. I’d say the biggest thing is I feel normal now. It’s just we still have to pump the breaks on everything, cause even though I feel good I’m still really not there yet.
		0012, Female: Pretty Good. I mean, it does suck that I have to stay isolated from everybody and everything. But it is nice to at least be one step closer to, to getting back into my normal life and being able to actually see people, have friends stop by of course, making sure that they’re yeah, nobody’s sick and they’re sanitizing and whatnot.
Staying busy after discharge	Using different resources to stay busy, mention of staying busy/distracted when discharged and out of the hospital	0004, Male: Oh. I just tried to keep busy.
		0006, Female: Having my smart phone and having the iPad. I have a Pinterest account. You’re a girl; I’m sure you’re familiar with Pinterest. I sat on Pinterest usually all day long, Facebook.
		0011, Male: I feel like blocking the day was what my mom and I did. And it was nice to have some sort of routine when we were in the hospital specifically. So, they want you to do so many labs in a day. So, we would break up our walking time. We’d watch Netflix for a little and then we’d go and do two or three laps and then we’d come back and eat lunch and then do this and then, go back out for a couple of labs.
Support system post HSCT	Any mention of friends and family being a coping/relief during and after transplant and after discharge	0003, Female: I would say talk with my daughter a lot.
		0012, Female: For coping, I mean, really all I did, I had my mom with me, so, we would just talk. She’s always been my support system.
		0020, Female: A lot of FaceTime. A lot of FaceTime, that helps. I call my son at least two or three times in a day just to talk to him. And my husband stops up on his breaks. So, yeah. Plus, I have my aunt and uncle here, and we’re always joking and talking. And we’re always together.
Social Networking	Interest or disinterest in social media platforms to form social connections	0003, Female: I have some on Instagram, and you just write back and forth with them. I think it’s My Cancer Patient or something on there, and a lot of people connect with everybody on there. We’ve done tell your stories and quite a few things and help each other out with all of it. And I’ve met a lot of people on there that actually have the same thing that I did.
		0016, Female: Probably just social media and being on my phone.
Physical activity	Discussion of using physical activity to cope post transplant and after discharge	0013, Male: I’ve done two scenic rides so far because Sarah, the nurse practitioner said maybe start with just the scenic ride so you can go slow and get your legs underneath you.
		0018, Male: Well, my stress reliever has always been the lifting. I have dropped belly fat. I no longer have a dad bod, so I’m trying and that’s always my main thing is, well how do I cope with stress is just weightlifting, working, and just being around friends and family. That’s really all you can do.
Reflection of Coping Methods	Any mention of changing or not changing coping methods while staying in hospital	0004, Male: Oh just keep in mind that you gotta stay busy while you’re in there. You got to keep your mind busy while you’re in there.
		0006, Female: No. I’m 31 years old. I’m young. I have two kids. So, of course I was stressed not being with them. But there’s nothing you can do. You just gotta go with the flow. I was scared at times, but that’s what Ativan is for. They give it to cancer patients. And if I was full of anxiety that day, I took an Ativan. You have to take advantage of the prescriptions that they provide for you.
**Theme 3: Mindfulness Activities**		
Mindfulness after hospitalization	Any discussion of using mindfulness after hospitalization and post treatment	0006, Female: I’m a high energy person, so I try practicing mindfulness. I did some Zoom calls with a psychologist, and she tried telling me about the breathing exercises.
		0015, Male: Meditate. It was pretty good. It’s very relaxing.
Useful strategy post HSCT	Timing of mindfulness to be most useful	0003, Female: I just think that I should probably do it again, actually, because it helped me a lot with my stress. But now that I’m with my daughter, I really don’t have any stress. In the hospital, I did, but now that I’m out and it’s over and everything’s alright, now I really—the scary part’s over with basically.
		0015, Male: I use it every once in a while, since we’re not going nowhere.
		0019, Male: For me, I would say after, but I guess it really depends on the person. When I was in the hospital, I wasn’t in the mood to do anything other than lay in my bed, and order food, and just watch TV, and just to bypass all this time. But after being out and being able to sort of get back to my life, I’ve thought about it before, but I haven’t really gotten to it, yet I guess.
Interest in mindfulness post HSCT	Interest or disinterest in mindfulness post discharge	0005, Male: I mean, I definitely think that just by—for example, earlier today when I started having that moment where I was kind of spiraling for a little bit, I definitely think that taking a moment to just focus on your breathing or something can really kind of slow that down and maybe help you snap out of it and not start downward spiraling.
		0014, Male: Yeah, it might just force me to sit there and really think through something without getting distracted or without moving onto something else before you really give something enough time.
		0014, Male: Probably less useful now that I’m kind of back on a more normal routine. But it’s tough to say. I’d say if treatment was completely done and everything was just moving forward, then I think I would be fine, but who knows.
Improve engagement post HSCT	Discussion of ways to improve engagement and attendance in a mindfulness program	0004, Male: The possibility of time, different times, scheduling.
		0002, Male: Well, mostly because I don’t—I don’t live out there. So, it’s, like, two–and–a–half–hour drive. But, you know, when I am there—I don’t know, I just—I’m afraid I’m going to run into a bunch of old people.
Mindfulness reflection	Any mention of mindfulness being a useful strategy as they reflect on cancer continuum	0003, Female: Oh, okay. It probably would help me, but I was just so nervous the whole time and it was just a lot to take in.
		0012, Female: Yeah. Well, when it comes to meditation and being self-aware of your problems and your emotions, it’s definitely important to first be aware. I’ve usually been the type to figure out ways around depression or anxiety usually just by staying optimistic. I definitely feel like it’d be like having the time to reflect whether it’s during meditation or yoga. Being able to put aside that that time for yourself is very important. I definitely would like to get into yoga. I think that would be healthy.

## Data Availability

Data is available from the corresponding author upon reasonable request.

## Supplementary Materials

The following supporting information can be downloaded at: https://www.mdpi.com/article/10.3390/cancers14112760/s1, Supplementary Information: Semi-structured interview guide.

## References

[1] National Cancer Institute Adolescents and Young Adults with Cancer. https://www.cancer.gov/types/aya.

[2] Smith A.W., Keegan T., Hamilton A., Lynch C., Wu X.-C., Schwartz S.M., Kato I., Cress R., Harlan L., AYA HOPE Study Collaborative Group (2018). Understanding care and outcomes in adolescents and young adults with cancer: A review of the AYA HOPE study. Pediatr. Blood Cancer.

[3] Mehta P.A., Rotz S.J., Majhail N.S. (2018). Unique Challenges of Hematopoietic Cell Transplantation in Adolescent and Young Adults with Hematologic Malignancies. Biol. Blood Marrow Transplant..

[4] Mathanda R.R., Hamilton B.K., Rybicki L., Advani A.S., Colver A., Dabney J., Ferraro C., Hanna R., Kalaycio M., Lawrence C. (2020). Quality-of-Life Trajectories in Adolescent and Young Adult versus Older Adult Allogeneic Hematopoietic Cell Transplantation Recipients. Biol. Blood Marrow Transplant..

[5] Saba N., Abraham R., Keating A. (2000). Overview of autologous stem cell transplantation. Crit. Rev. Oncol..

[6] Molassiotis A., Morris P. (1997). Suicide and suicidal ideation after marrow transplantation. Bone Marrow Transplant..

[7] Wolcott D.L., Fawzy F.I., Wellisch D.K. (1986). Psychiatric aspects of bone marrow transplantation: A review and current issues. Psychiatr. Med..

[8] Belec R.H. (1992). Quality of life: Perceptions of long-term survivors of bone marrow transplantation. Oncol. Nurs. Forum.

[9] Jenkins P.L., Linington A., Whittaker J., Path F. (1991). A Retrospective Study of Psychosocial Morbidity in Bone Marrow Transplant Recipients. J. Psychosom. Res..

[10] Horton-Deutsch S., Day P.O., Haight R., Babin-Nelson M. (2007). Enhancing mental health services to bone marrow transplant recipients through a mindfulness-based therapeutic intervention. Complement. Ther. Clin. Pract..

[11] Mehta R.S., Rezvani K. (2016). Immune reconstitution post allogeneic transplant and the impact of immune recovery on the risk of infection. Virulence.

[12] Creswell J.D. (2017). Mindfulness Interventions. Annu. Rev. Psychol..

[13] Peterson L.G., Pbert L. (1992). Effectiveness of a meditation-based stress reduction program in the treatment of anxiety disorders. Am. J. Psychiatry.

[14] Cladder-Micus M.B., Speckens A.E., Vrijsen J.N., Donders A.R.T., Becker E.S., Spijker J. (2018). Mindfulness-based cognitive therapy for patients with chronic, treatment-resistant depression: A pragmatic randomized controlled trial. Depress. Anxiety.

[15] Knill K., Warren B., Melnyk B., Thrane S.E. (2021). Burnout and Well-Being: Evaluating Perceptions in Bone Marrow Transplantation Nurses Using a Mindfulness Application. Clin. J. Oncol. Nurs..

[16] Compernolle M.C., Sledge J.A. (2020). Effects of a Mindfulness Intervention on Hospitalized Patients With Hematologic Malignancies and Their Caregivers. Oncol. Nurs. Forum.

[17] Donovan E., Martin S.R., Seidman L.C., Zeltzer L.K., Cousineau T.M., A Payne L., Trant M., Weiman M., Knoll M., Federman N.C. (2019). A Mobile-Based Mindfulness and Social Support Program for Adolescents and Young Adults With Sarcoma: Development and Pilot Testing. JMIR mHealth uHealth.

[18] Van Der Gucht K., Takano K., Labarque V., Vandenabeele K., Nolf N., Kuylen S., Cosyns V., Van Broeck N., Kuppens P., Raes F. (2017). A Mindfulness-Based Intervention for Adolescents and Young Adults After Cancer Treatment: Effects on Quality of Life, Emotional Distress, and Cognitive Vulnerability. J. Adolesc. Young-Adult Oncol..

[19] Pathrose S.P., Everett B., Patterson P., Ussher J., Salamonson Y., McDonald F., Biegel G., Ramjan L. (2021). Mindfulness-Based Interventions for Young People With Cancer. Cancer Nurs..

[20] Lewis A., Oppenheim A. (1994). Questionnaire Design, Interviewing and Attitude Measurement, London, Pinter. Pp 303. £14.99 paperback, £39.50 hardback. J. Community Appl. Soc. Psychol..

[21] Braun V., Clarke V. (2006). Using thematic analysis in psychology. Qual. Res. Psychol..

[22] Dedoose Version 9.0.17. A Web Application for Managing, Analyzing, and Presenting Qualitative and Mixed Method Research Data. www.dedoose.com.

[23] Vinci C., Pidala J., Lau P., Reblin M., Jim H. (2020). A mindfulness-based intervention for caregivers of allogeneic hematopoietic stem cell transplant patients: Pilot results. Psycho-Oncology.

[24] Vinci C., Reblin M., Jim H., Pidala J., Bulls H., Cutolo E. (2018). Understanding preferences for a mindfulness-based stress management program among caregivers of hematopoietic cell transplant patients. Complement. Ther. Clin. Pract..

[25] Grossman P., Zwahlen D., Halter J.P., Passweg J.R., Steiner C., Kiss A. (2014). A mindfulness-based program for improving quality of life among hematopoietic stem cell transplantation survivors: Feasibility and preliminary findings. Support. Care Cancer.

[26] Bauer-Wu S., Sullivan A.M., Rosenbaum E., Ott M.J., Powell M., McLoughlin M., Healey M.W. (2008). Facing the Challenges of Hematopoietic Stem Cell Transplantation With Mindfulness Meditation: A Pilot Study. Integr. Cancer Ther..

[27] Duong N., Davis H., Robinson P.D., Oberoi S., Cataudella D., Culos-Reed S.N., Gibson F., Gotte M., Hinds P., Nijhof S.L. (2017). Mind and body practices for fatigue reduction in patients with cancer and hematopoietic stem cell transplant recipients: A systematic review and meta-analysis. Crit. Rev. Oncol. Hematol..

