# Supplementary material for: Mindfulness in Adolescent and Young Adult (AYA) Patients Undergoing Hematopoietic Stem Cell Transplantation (HSCT): A Qualitative Study
Source: Cancers (Basel). 2022 Jun 2;14(11):2760. doi: 10.3390/cancers14112760 (PMC9179916; doi:10.3390/cancers14112760)
Supplement: Supplementary file 1 [file cancers-14-02760-s001.zip › cancers-1715788-supplementary.pdf]

Mindfulness in Adolescent and Young Adult (AYA) Patients Undergoing Hematopoietic Stem Cell Transplantation (HSCT): A Qualitative Study

Sylvia L. Crowder, Rachel Sauls, Laura Redwine, Michael Nieder, Omar Albanyan, Hassaan Yasin, Farhad Khimani, Marilyn Stern

Supplemental Information: Semi-structured interview guide

Interview Questions (*n* = 20)

- Confirm quiet location;
- Get consent to begin recording;
- Start recording.

Time 1 (Prior to HSCT)

Thank you for taking the time to talk with me today about your experiences as a patient.

As we described in the consent form you signed, we are in the process of developing a program to help allogeneic hematopoietic cell transplant patients better manage their stress during the initial hospitalization period, before the procedure, and then before discharge and then again 90 days-post hospitalization. We'd like to ask you several questions about your experiences so far, and what may be useful for you to help manage stress. I will be describing several strategies we are thinking about including in the program we are developing, and we are interested in your opinion about these.

Do you have any questions before we begin?

Please be aware that I will be audiotaping the session so I don't miss any information you give us.

General Questions

1. How have you been feeling lately?
2. For your upcoming hospitalization, what do you think it is going to be like for you? How are you feeling emotionally? *Note:* If they say a specific emotion ask what it looks like (e.g., what does it mean being sad for you? what does being sad looks like for you?)
    a. *Probe: Are you feeling angry, sad, hopeful, scared?*
    b. *Probe: How much stress are you experiencing?*
3. What are some ways you cope with stress and [insert negative emotions described by the patient?
    a. How do you manage stress at home compared to the hospital?
    b. When you have dealt with difficult situations before, such as at home, how is that different or similar to what you are experiencing right now?
4. What has helped you the most? Why?
    a. *If they respond with something like thinking positively or similar, ask*: "Positive thinking can mean different things to different people. How did you think positively? How did that help you?
5. How do you envision what life is going to be like after this procedure is completed and you are discharged? *Note:* If they do not know, ask how they cope with the uncertainty.
6. What does your social support look like? (i.e. Family, friends, what kind of social network do you have)
    a. How do they support you?

7. Would you be interested in a way to connect with others via video chat or some sort of other social networking platform?
    a. What type of social media do you use most frequently?
    b. How likely is that you would use a social media networking site -where you might be able to connect with others- dedicated to these issues?

Mindfulness Questions

As I mentioned, we are in the process of developing a program to help patients better manage their stress during the initial hospitalization period, as well as the 90 days-post hospitalization. We are thinking about developing a mindfulness-based program.

8. Have you ever heard of mindfulness before?
9. When you hear the word mindfulness, what does it mean to you?

A common definition of mindfulness is the ability to stay focused on the present moment without judging whether it is good or bad. This basically means that a participant in our program would learn skills to help keep them focused, help manage stress and make clearer decisions.

10. Tell me your general thoughts about what I just described.
    a. *Prompt: How do you think what I just described would help with stress?*
11. Have you ever practiced anything related to mindfulness before or any other stress reduction strategy related to mindfulness?
    a. *If yes, ask: What are some things you have done (e.g., Yoga/massage/color therapies etc.)? What have you liked?*
    b. *If no, ask: Is there any special reason why you haven't tried any of these practices before?*
12. How useful do you think this type of strategy would be for you?
13. How willing are you to try what I just described?
    a. *Prompt: If not willing, what changes would you make?*

For our program we are using what is called Mindfulness meditation, that is a mental training practice that involves focusing your mind on your experiences (like your own emotions, thoughts, and sensations) in the present moment. Mindfulness meditation can involve breathing practice, mental imagery, awareness of body and mind, and muscle and body relaxation.

14. Have you ever meditated before?
    a. *If yes, ask: What types of meditation have you tried? How many times have you tried them?*

In our mindfulness program, guided meditation would last for about 15 to 20 minutes per day, and you would be asked to focus on something specific for that period of time. For instance, you may focus only on your breathing or only on the sensations you feel in your body.

15. Tell me your general thoughts about the strategy I just described.
    a. *Prompt: How do you think what I just described would help with stress?*
    b. How useful do you think this type of strategy will be for you?
16. How willing would you be to participate in a mindfulness program including meditation as I just described, here in Moffitt?
    a. *If no, ask: Would you be more willing to participate if someone like another patient, a doctor, or a nurse recommended the program?*
17. What could be some barriers to engaging in a mindfulness-based stress management program?

It is recommended to get out of bed and move around as part of the recovery process afterwards. How do you feel about that?

18. What type of physical activities will motivate you in recovery (i.e., yoga, walking)?
19. Would you say that you were physically active before this process started?
20. What could be some barriers to engaging in physical activities (i.e., pain, fatigue, lack of motivation)?

Time 2: Immediately Following HSCT:

Thank you for taking the time to talk with me today about your experiences with your recent transplant.

1. I know you recently received the transplant, how are you feeling? How have you been since we last talked?
2. How were you feeling emotionally during the weeks surrounding your transplant procedure?
    a. *Probe: Were you feeling angry, sad, hopeful, scared?*
    b. *Probe: How much stress did you experience?*
3. How did you mentally prepare for this procedure? Did you use any of the information that I shared with you when we met the first time?
4. What did you do to cope with stress and emotions during the transplant procedure?
5. Of the different ways you tried to cope [list ways], what helped you the most? Why?
    a. *If they respond with something like thinking positively or similar, ask*: "Positive thinking can mean different things to different people. How did you think positively? How did that help you?
6. Looking back now, are there things you wished you had done differently to manage [insert negative emotions]? *Note:* If they do not mention any negative emotion you could say "to manage stress".
7. *What are you doing now to cope with your emotions and stress and what do you think you will do in the future to cope with emotions and stress?*

Mindfulness Questions

As I mentioned before your HCT, we are in the process of developing a program to help patients better manage their stress during the hospitalization period, and up to 90 days-post hospitalization. We are thinking about developing a mindfulness-based program.

8. I know in the first interview we spoke a little about Mindfulness, since then has your interpretation of mindfulness changed?
9. Did you practice mindfulness since the beginning of your treatment?
    a. *If yes ask: What are some things you have done?*
    b. *What did you take advantage of and what did you like from the "integrative medicine consult" while inpatient (Yoga/massage/color therapies etc.)*

As a reminder, mindfulness is the ability to stay focused on the present moment without judging if it is good or bad. This basically means that a participant in our program would learn skills to help keep them focused on the present moment in order to help manage stress and make clearer decisions.

10. In our first meeting you told me your thoughts about this description, but how do you feel about mindfulness after the transplant procedure?
    a. *Prompt: How do you think what I just described would help with the stress of the transplant procedure?*
11. How useful do you think this type of strategy would have been for you during hospitalization before the procedure?
12. How useful would it be for you now?
13. How willing would you be to try what I just described?
    a. *Prompt: If not willing, what changes would you make?*

As we discussed in our first meeting, part of this program would involve a daily meditation to help with managing stress.

14. Do you feel or think the same way about meditation than when we talked the first time? Do you think of it in any other or different way? What is different this time?
15. Have you meditated during your hospitalization?
    a. *If yes, ask: What are some things you have done?*

As you know, in our mindfulness program, meditation would last for about 20 minutes per day, and you would be asked to focus on something specific for that period of time. For instance, you may focus only on your breathing or only on the sensations you feel in your body.

16. Do you feel or think the same way about this strategy than when we first talked? Do you think of it in any other or different way? What is different this time?
    a. *Prompt: How do you think what I just described would help with stress?*
17. How useful do you think this type of strategy would be for you?
18. How willing would you be to try what I just described?
    a. *Prompt: If not willing, what change would you make?*
19. What type of information would increase your chances of entering a mindfulness-based stress management program? In other words, is there anything that would make you more likely to participate in this program?
    a. *Probe: Sometimes people need to talk with someone else who went through the program or see data showing that the program works. Other people may want their doctor or nurse to recommend the program, or even their patient. Would any of these things increase your chances of participating?*
20. How your social network (e.g., family, friends) have supported you during and after the transplant procedure?
21. Is there anything else you would like to talk about mindfulness?
22. Do you have any other question about mindfulness or meditation at this time?

Final questions:

In our first meeting we talked about different ways to connect with others via video chat or some other social networking platform.

23. Would find a social chat room with friends and family and other patients undergoing similar procedures helpful?

24. Have you ever felt socially isolated during your hospitalization? Do you think that using a social network platform could help with it?

<u>Time 3: Three Months Post HSCT:</u>

Thank you for taking the time, once more, to talk with me today about your experiences with your recent transplant.

1. I know you recently were discharged, how are you feeling? How have you been since we last talked?
2. How are you feeling emotionally?
    a. *Probe: Are you feeling angry, sad, hopeful, scared?*
    b. *Probe: How much stress are you experiencing?*
3. What did you do to cope with stress and emotions during the hospitalization?
4. Of the different ways you tried to cope [list ways], what helped you the most? Why?
    a. *If they respond with something like thinking positively or similar, ask*: "Positive thinking can mean different things to different people. How did you think positively? How did that help you?

5. Looking back now, are there things you wished you had done differently to manage [insert negative emotions]?
6. Now tell me about what life is like after hospital discharge. Are you staying at an apartment or Hope Lodge? What is this time period like for you?
7. What are you doing now to cope with your emotions and stress and what do you think you will do in the future to cope with emotions and stress?
8. Overall, what do you think you have gained as a person and/or emotionally from going through the HCT?

Mindfulness Questions

9. I know in the first and second interview we spoke about Mindfulness, since then has your interpretation of mindfulness changed? Have you practice it?
10. Since you were discharged from the hospital have you used any mind-body therapies, such as meditation, yoga, massage? Has it been helpful?

I'd like to hear your feedback now that you have been discharged from hospitalization about how useful mindfulness and mindfulness meditation maybe for you.

USE ONLY IF YOU FEEL THE PATIENT MAY NEED A REMINDER IF NOT FOLLOW TO THE QUESTIONS: As a reminder, mindfulness is the ability to stay focused on the present moment without judging if it is good or bad. This basically means that a participant in our program would learn skills to help keep them focused on the present moment in order to help manage stress and make clearer decisions.

11. How do you think mindfulness and meditation would help you to manage stress?
12. Have you tried any mindfulness program or meditated things the last time we spoke?
13. How useful do you think this type of strategy would have been for you during hospitalization?
14. How useful would it be for you now? How willing would you be to try it?
    a. *If not useful or willing, ask: What changes would you make?*
15. What type of information would increase your chances of entering a mindfulness-based stress management program? In other words, is there anything that would make you more likely to participate in this program?
    a. *Probe: Sometimes people need to talk with someone else who went through the program or see data showing that the program works. Other people may want their doctor or nurse to recommend the program, or even their patient. Would any of these things increase your chances of participating?*

Tele-health Questions

16. How do you feel about telehealth appointments with your doctor?
17. Do you prefer telehealth visits or in-person visits? And why?
18. How would you want to improve your telehealth visit with your physician?
19. What do you think about a telehealth delivery of a mindfulness intervention?
20. What do you think might be the benefits of having a mindfulness intervention remotely?

Final questions:

In our second meeting we talked about different ways to connect with others via video chat or some other social networking platform.

21. Since the last time we talked, have you used any social media networking site to connect with others dedicated to these issues?
    a. If yes, ask: What platforms have you used? Has it been helpful?
    b. If no, ask: How likely would you be to use a social media networking site – where you might be able to connect with others/dedicated to these issues?
22. What are some barriers that you think it could prevent other people to use these platforms as a support resource?
23. As we have talked about before, we are planning to develop an intervention that would include a networking mechanism, or ways to communicate with other patients undergoing similar treatments. We are wondering to what extent this might be something you would be interested in?
